# Sex differences in voluntary running behavior between C57BL/6 and BALB/cJ mouse strains do not correspond to changes in VO2 and RER

**DOI:** 10.14814/phy2.70604

**Published:** 2025-10-10

**Authors:** N. Amiri, H. Zhang, R. McMillan, R. W. Grange, J. C. Drake

**Affiliations:** ^1^ Department of Human Nutrition, Foods, and Exercise Virginia Tech Blacksburg Virginia USA; ^2^ Institute for Critical Technology and Applied Science Virginia Tech Blacksburg Virginia USA

**Keywords:** exercise, RER, sexual dimorphism, VO2

## Abstract

Exercise adaptations are influenced by sex and genetic background, contributing to variability in metabolic and physiological responses. This study investigates sex and strain‐specific differences following voluntary wheel running exercise training in submaximal and maximal oxygen consumption (VO2), respiratory exchange ratio (RER), and body composition in C57BL/6 and BALB/cJ mice. Male and female mice underwent 4 weeks of voluntary wheel running, followed by sub‐maximal and maximal treadmill tests in a metabolic chamber. Results indicate differences in running volume across sexes and strains did not consistently predict changes in physiological adaptations. Female C57BL/6 mice, which ran three times more than males on average, exhibited higher submaximal VO2 compared to their male counterparts and to female BALB/cJ mice, despite no differences in RER. In contrast, male BALB/cJ mice, which ran nearly double the distance of their female counterparts, showed a modest decrease in average RER. During maximal treadmill tests, male BALB/cJ mice demonstrated enhanced endurance capacity, characterized by increased distance run and a trend towards lower RER and blood lactate levels at exhaustion, despite no significant changes in VO2 max. Regarding body composition, female C57BL/6 mice experienced a reduction in fat mass and an increase in lean mass, whereas no significant changes were observed in BALB/cJ mice of either sex. The study highlights the need to consider sex‐ and strain‐specific factors when evaluating metabolic and endurance adaptations, and suggests VO_2_ testing in mice may not reflect adaptive response to voluntary wheel running.

## Graphical abstract

Study and results overview.

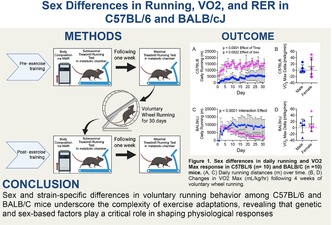

## 1 INTRODUCTION

The advantageous effects of exercise in preventing pathologies such as obesity, cardiovascular disease, and diabetes are widely recognized (Rockl et al., 2007). However, understanding the mechanisms behind differences in adaptive responses to exercise in humans can be complex due to genetic, environmental, and lifestyle factors. To understand this complexity, animal models, such as mice, are commonly used to manipulate genetic and environmental variables, providing insights that may be translated to human health (Voss et al., 2013). Previous studies in humans have demonstrated that males and females respond differently to various forms of exercise, which may be attributed to inherent physiological and metabolic differences between the sexes (Miller et al., 1993).

For humans, sex‐based differences in muscle performance are often observed. Males generally have larger muscle fiber cross‐sectional area (CSA), but specific force is equivalent between sexes when normalized to CSA (Bartolomei et al., 2021; Miller et al., 1993). Meta‐analytic data show that men have larger CSA across all fiber types and a higher proportion and area of fast‐twitch (Type II) fibers, while women exhibit greater relative proportions and area percentages of slow‐twitch (Type I) fibers, which may contribute to superior fatigue resistance and endurance capacity in females (Nuzzo, 2024). In rodents, female mice are reported to be relatively protected from force loss and contraction‐induced damage in dystrophic and aging models (Hourdé et al., 2013), suggesting potential sex‐specific protective mechanisms. These differences can be influenced, in part, by hormonal variations, particularly the effects of estrogen and testosterone on muscle and metabolic function, which in turn affect oxygen consumption (VO2) and respiratory exchange ratio (RER) (Holcomb et al., 2022; Massett et al., 2021). Although rodent models are commonly utilized for studying exercise adaptation and behavior, there are relatively few longitudinal studies on physiological differences between sexes.

Genetic background of mice used in exercise research is also a contributing factor to our understanding of adaptive responses to exercise. Lerman et al. demonstrated significant variability in both forced and voluntary endurance exercise performance among seven different inbred mouse strains; specifically, Swiss Webster (SW) and FVB/NJ mice exhibited superior performance in treadmill‐based (forced) endurance exercise tests, while C57BL/6 mice performed the poorest (Lerman et al., 2002). Interestingly, despite their poor performance in forced treadmill exercise, C57BL/6 mice displayed the highest levels of voluntary wheel‐running activity compared to strains such as SW, FVB/NJ, and DBA, emphasizing that forced exercise performance does not necessarily predict voluntary exercise behavior. Supporting this strain‐dependent variability, Masset et al. highlighted considerable differences in exercise training responses across inbred and hybrid mouse strains (Massett & Berk, 2005). They reported endurance performance enhancements, measured as increased distance run, run time, and work performed during treadmill tests, were five to seven times greater in FVB mice compared to C57BL/6 and BALB/cJ mice. These findings underline the significant impact of genetic background on both forced exercise performance and training adaptations, further reinforcing the need to consider strain‐specific differences when evaluating exercise outcomes (Massett & Berk, 2005).

Furthermore, strain‐specific physiological characteristics may also influence the adaptive response to exercise. C57BL/6 mice are commonly used in metabolic research due to their predisposition to obesity and diabetes under certain dietary conditions, a characteristic linked to their susceptibility to diet‐induced insulin resistance and hyperglycemia. Therefore, this makes them a valuable model for studying metabolic syndrome and type 2 diabetes (Surwit et al., 1988). Conversely, BALB/cJ mice are frequently utilized in immunological studies due to their robust and consistent immune responses, particularly in generating strong antibody responses. These mice exhibit distinct metabolic profiles characterized by lower susceptibility to obesity under similar dietary conditions compared to C57BL/6 mice, potentially due to differences in energy expenditure and immune–metabolic interactions (Zhang et al., 2023). These physiological differences make the two strains suitable for addressing specific research questions related to metabolism and immunity. Observations from comparative studies on strain‐specific metabolism and immune responses form the basis for these distinctions. DBA/1 J and SW mice display significantly greater cardiac contractility, while BALB/cJ mice exhibit reduced cardiac function (Lerman et al., 2002). Despite these insights, it remains unclear how these factors interact between sexes within the same strain.

Our aim here was to explore longitudinal differences in response to voluntary exercise training using two different strains of mice that have been reported to have different voluntary running behaviors, C57BL/6 and BALB/cJ mice, utilizing both male and female animals. We performed sub‐maximal and maximal treadmill running tests in a metabolic chamber, as well as body composition assessments, before and after 1 month of voluntary wheel running to model aerobic exercise training (Brisendine et al., 2024; Drake, Wilson, Cui, et al., 2021; Laker et al., 2017; Nwadike et al., 2018). Our findings shed light on the relationship between running behavior across sex and genetic background and the adaptive response to exercise training.

## 2 MATERIALS AND METHODS

### 2.1 Animals

**FIGURE 1:**
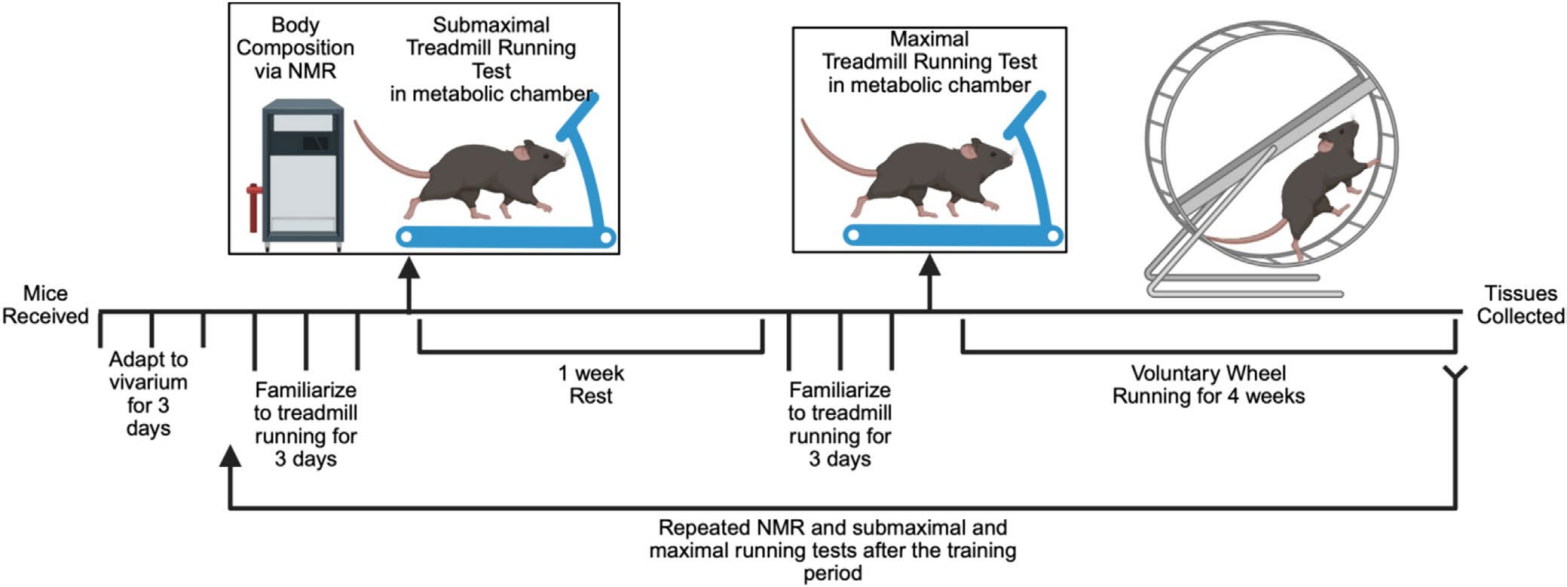
Experimental Design. Body composition was analyzed in conscious mice by nuclear magnetic resonance (NMR) 1 day prior to beginning treadmill testing and after 30 days of exercise training. Following 3 days of familiarization to treadmill running and 1 day after the NMR measurements, mice underwent the first submaximal treadmill running test. Following a one‐week recovery period, mice were re‐familiarized with treadmill running and then underwent a maximal treadmill running test. Mice were individually placed in running wheel cages and allowed to run ad libitum for 4 weeks. After 4 weeks, body composition and treadmill testing were repeated while maintaining access to running wheels during the posttesting phase.

All experimental procedures were approved by Virginia Polytechnic Institute and State University Institutional Animal Care and Use Committee. Male and female C57BL/6 and BALB/cJ mice were obtained commercially at 8 weeks of age (Jackson Laboratories, Bar Harbor, ME; strain #000664 and #000651, respectively). Mice were fed the Teklad Global 18% Protein Rodent Diet (2018, Envigo, Madison, WI) throughout the study. Mice were acclimated for 1 week prior to study onset in temperature‐controlled quarters (22°C) on a 12:12 h light–dark cycle with ad libitum access to food and water. Overall study design is illustrated in Figure 1.

### 2.2 Body composition

Nuclear magnetic resonance (NMR) spectroscopy was used to assess body composition (Bruker Minispec). Following a system calibration to the manufacturer's standards, mice were placed in a restrainer tube, placed into the sample chamber, and scanned (<2 min).

### 2.3 Treadmill testing

Mice underwent two separate acute exercise tests designed to test submaximal and maximal exercise performance before and after 4 weeks of voluntary wheel running (i.e., exercise training). The software collects cumulative running distance and timestamps at a default recording interval of 2 min. Both exercise tests were performed on a modular treadmill enclosed with a metabolic chamber (Oxymax; Columbus Instruments, Columbus, OH) with a 5‐degree incline and were overseen by the same researcher. Mice were acclimated to treadmill running for 3 days prior to testing, which consisted of running on a treadmill at 10 m/min at a 0‐degree incline for 10 min, as previously described (Brisendine et al., 2024; Drake, Wilson, Laker, et al., 2021; Laker et al., 2017). On the day of testing, mice were placed in the metabolic treadmill cages and allowed to acclimatize for 30 min prior to testing. During both the submaximal and maximal tests, a low‐intensity electrical stimulus (shock grid) located at the rear of each lane was used as a motivational tool to encourage running. The stimulus was minimal and applied only when mice failed to maintain pace, in accordance with approved animal care protocols. To minimize circadian variability, all submaximal and maximal VO_2_ tests were conducted between 9:00 a.m. and 12:00 p.m. Oxygen consumption (VO2) and respiratory exchange ratio (RER) were measured continuously during both tests by indirect calorimetry. Both tests were conducted before and after 30 days of voluntary wheel running, described below. *Submaximal exercise test*: Mice ran for a total of 90 min, which consisted of 10 min at 13 m/min, 10 min at 16 m/min, 50 min at 19 m/min, and 20 min at 21 m/min, as previously used to assess acute exercise‐related signaling (Drake, Wilson, Laker, et al., 2021; Laker et al., 2017). All mice completed submaximal exercise tests. *Maximal exercise test*: 7 days following submaximal testing, mice were re‐familiarized with the treadmill as before and maximal exercise performance was assessed on day four. Maximal exercise testing consisted of running at 5 m/min for 2 min with speed increasing by 5 m/min every 2 min thereafter until exhaustion was perceived. Exhaustion was defined as the point at which a mouse remained on the shock grid for more than 3 to 5 consecutive seconds without attempting to reengage in running, despite the motivational stimuli. At this point, the test was terminated, and the mouse was immediately removed from the treadmill. This criterion was used consistently across all animals to determine the point of volitional exhaustion. Blood lactate was assessed from the tail vein immediately before and after to confirm perceived exhaustion (more below). Running economy was calculated as average VO_2_ consumption × body weight (kg) × distance (m). Average VO_2_ for economy calculated was measured during the submaximal exercise test at a constant speed of 19 m/min for 50 min.

### 2.4 Blood lactate

Blood lactate concentrations were measured before and immediately after the exercise protocols via tail snip, as previous (Brisendine et al., 2024; Drake, Wilson, Cui, et al., 2021; Guan et al., 2024; Laker et al., 2017). Briefly, a small incision was made at the distal end of the tail, and a drop of whole blood was collected and analyzed using a handheld lactate analyzer (Nova Biomedical). All measurements were conducted within 15 s of blood collection to ensure accuracy. The tail was cleaned with 70% ethanol before and after sampling, and styptic powder was applied to stop the bleeding once the required sample was collected.

### 2.5 Voluntary wheel running

Following baseline assessments of body composition and sub‐maximal and maximal exercise performance, mice were individually housed in custom cages equipped with running wheels for 6 weeks total. Wheel revolutions were continuously recorded using magnetic counters, and total daily running distances were recorded via computer monitoring. After 4 weeks of undisturbed voluntary wheel running, postintervention assessments were conducted. Wheels were locked the evening prior to each postsubmaximal and maximal exercise test. Because posttesting could alter subsequent voluntary running behavior, daily running data were only reported for the uninterrupted 4‐week period.

### 2.6 Statistics

Data are presented as mean ± SD and were analyzed and plotted using GraphPad Prism (v10.1.0). Data were analyzed via Student's *t*‐test when one variable was present, repeated measures two‐way ANOVA when two variables were measured in the same animals over time. Posthoc analyses were only performed when a significant interaction between a categorical and quantitative variable was found, which is indicated in the figures where applicable. Significance was established a priori as *p* < 0.05.

## 3 RESULTS

**FIGURE 2:**
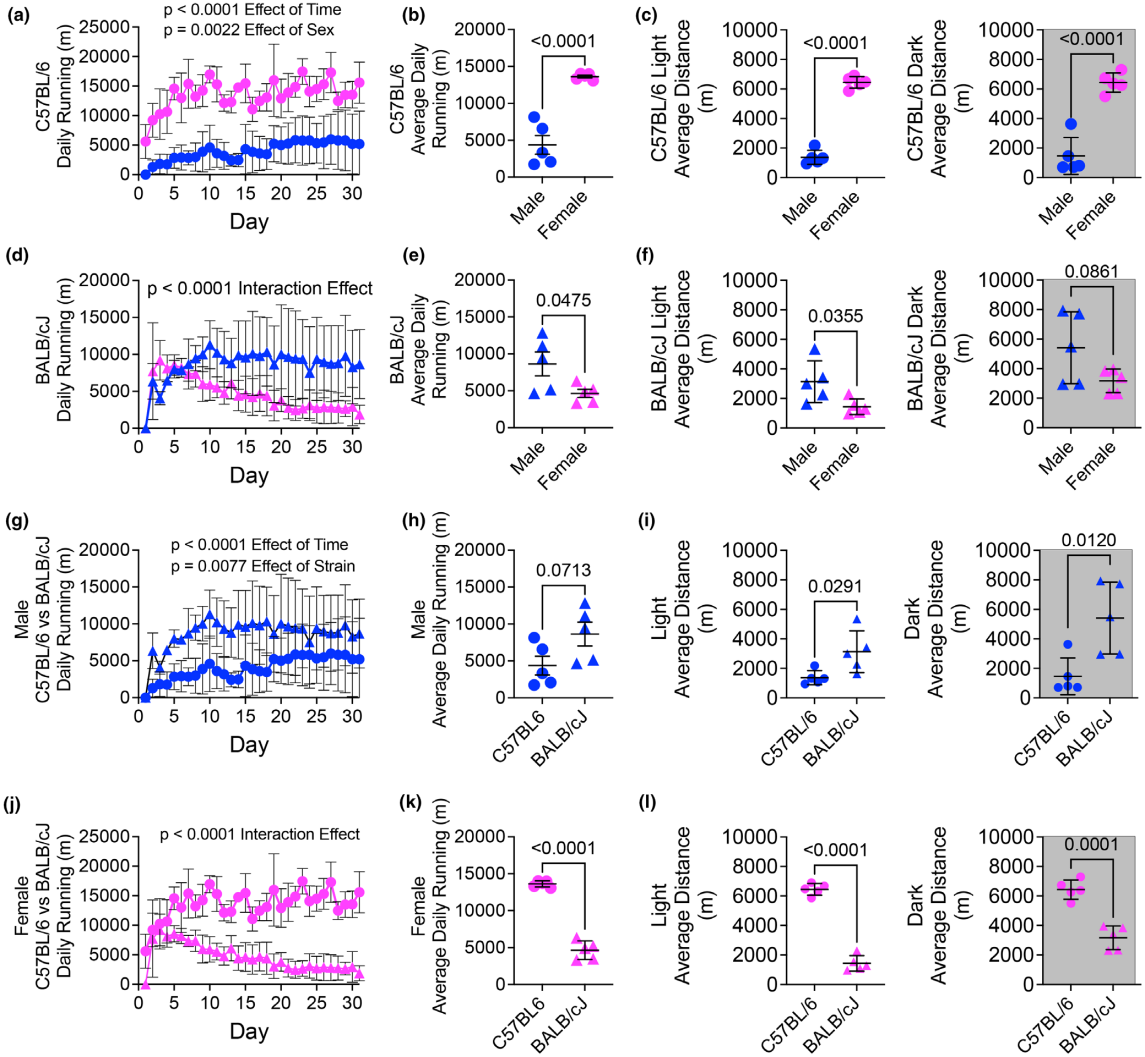
Sex‐ and strain‐specific differences in volitional running behavior during the training period. (a) Daily running distance of male and female C57BL/6 mice over the 30‐day training period (two‐way repeated measures ANOVA). (b) Average daily running distance across the training period in C57BL/6 mice (unpaired Student's *t*‐test). (c) Light and dark cycle analysis of C57BL/6 mice (unpaired Student's *t*‐test). (d) Daily running distance in BALB/cJ (two‐way repeated measures ANOVA). (e) Average daily running distance in BALB/cJ mice across the training period (unpaired Student's *t*‐test). (f) Light and dark cycle running distance for BALB/cJ mice (unpaired Student's *t*‐test). (g–i) Comparison of daily running distance and light/dark cycle behavior between male C57BL/6 and BALB/cJ mice (two‐way repeated measures ANOVA; unpaired Student's *t*‐test). (j–l) Comparison of daily running distance and light/dark cycle behavior between female C57BL/6 and BALB/cJ mice (two‐way repeated measures ANOVA; unpaired Student's *t*‐test).

Both male and female C57BL/6 mice increased volitional running volume over the course of the training period (Figure 2a). However, female C57BL/6 mice ran significantly more daily and on average over the entire training period than their male counterparts, which equated to female C57BL/6 mice running approximately three times more than male C57BL/6 mice (Figure 2a,b). This sex difference was observed across both the light and dark cycles, with females running significantly more during each phase (Figure 2c). BALB/cJ mice displayed different running behavior, with male BALB/cJ mice running more daily and on average over the entire training period compared to female BALB/cJ mice (Figure 2d,e). In contrast to C57BL/6 females, BALB/cJ female mice ran progressively less each day during the training period (Figure 2d). Also, analysis of light and dark cycle behavior revealed that male BALB/cJ mice ran more than females during both phases, although the difference in the dark cycle did not reach statistical significance (Figure 2d–f). Male BALB/cJ mice ran more compared to C57BL/6 male mice (Figure 2g,h). In contrast, female BALB/cJ mice ran significantly less than C57BL/6 female mice (Figure 2j–l), with female C57BL/6 increasing running volume over the course of the training period and female BALB/cJ mice decreasing running volume (Figure 2j).

**FIGURE 3:**
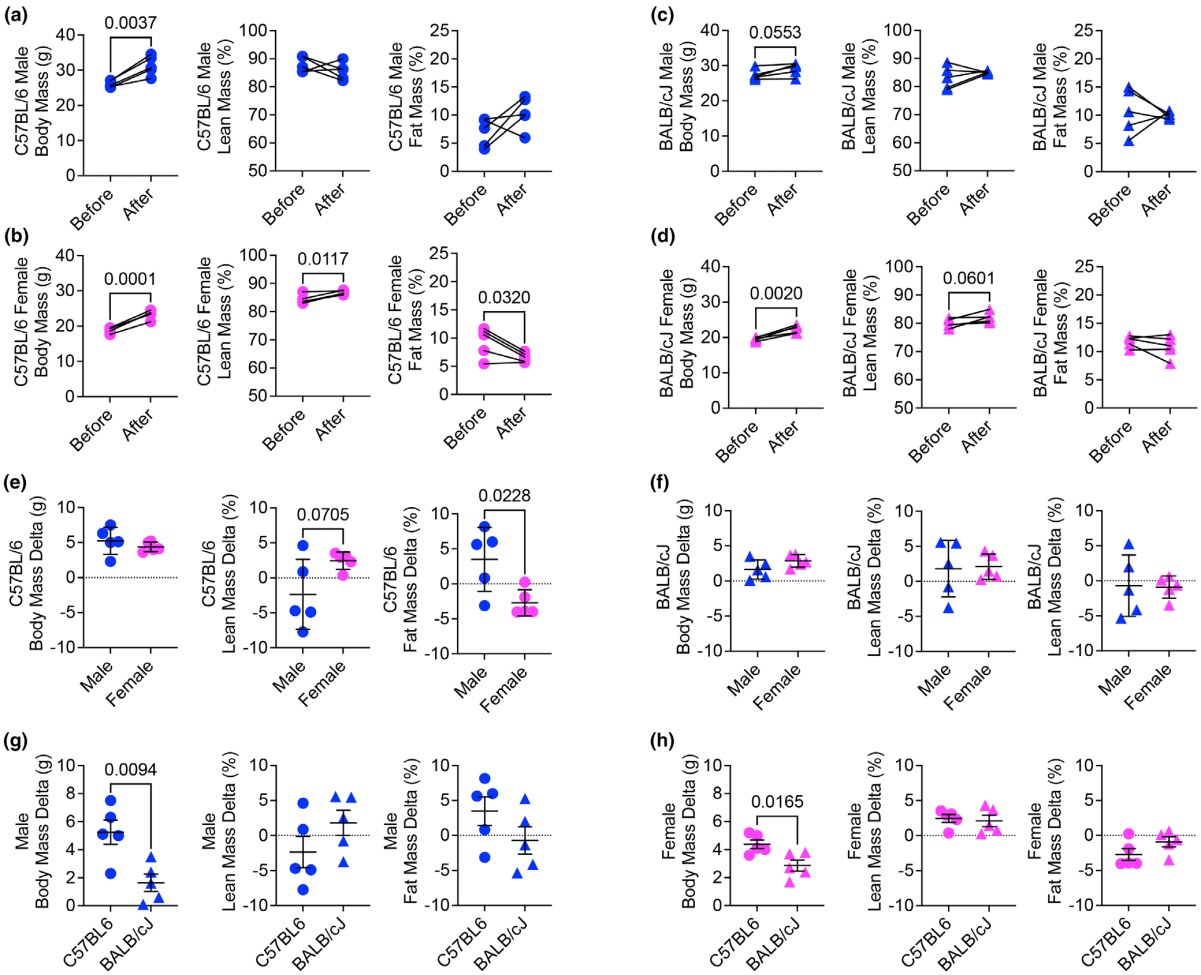
Strain and sex‐specific effects of voluntary exercise training on body mass and composition. (a–d) Changes in body mass and composition over the training period between male and female C57BL/6 and BALB/cJ mice. (e) Comparison of body composition between male and female C57BL/6 mice. (f) Comparison of body composition between male and female BALB/cJ mice. (g, h) *Co*mparisons between sexes across C57BL/6 and BALB/cJ strains. Student's *t*‐test was used for all comparisons.

Over the course of the training period, body mass increased in male C57BL/6 mice, as well as both female C57BL/6 and BALB/cJ mice, while male BALB/cJ tended (*p* = 0.0553) towards elevated body mass over the training period (Figure 3a–d). Female C57BL/6 mice increased lean mass and decreased fat mass during the training period, but no such changes were observed in male C57BL/6 mice (Figure 3a,b). No significant changes in body composition over the training period were observed in BALB/cJ mice (Figure 3c,d). While there was no difference in body mass between male and female C57BL/6 mice with voluntary exercise training, females had significantly less fat mass compared to males (Figure 3e). No differences in body composition between male and female BALB/cJ mice were found (Figure 3f). Although both male and female C57BL/6 mice had more body mass compared to respective BALB/cJ mice, no differences were found in body composition between same sexes across the two strains with voluntary running (Figure 3g,h).

**FIGURE 4:**
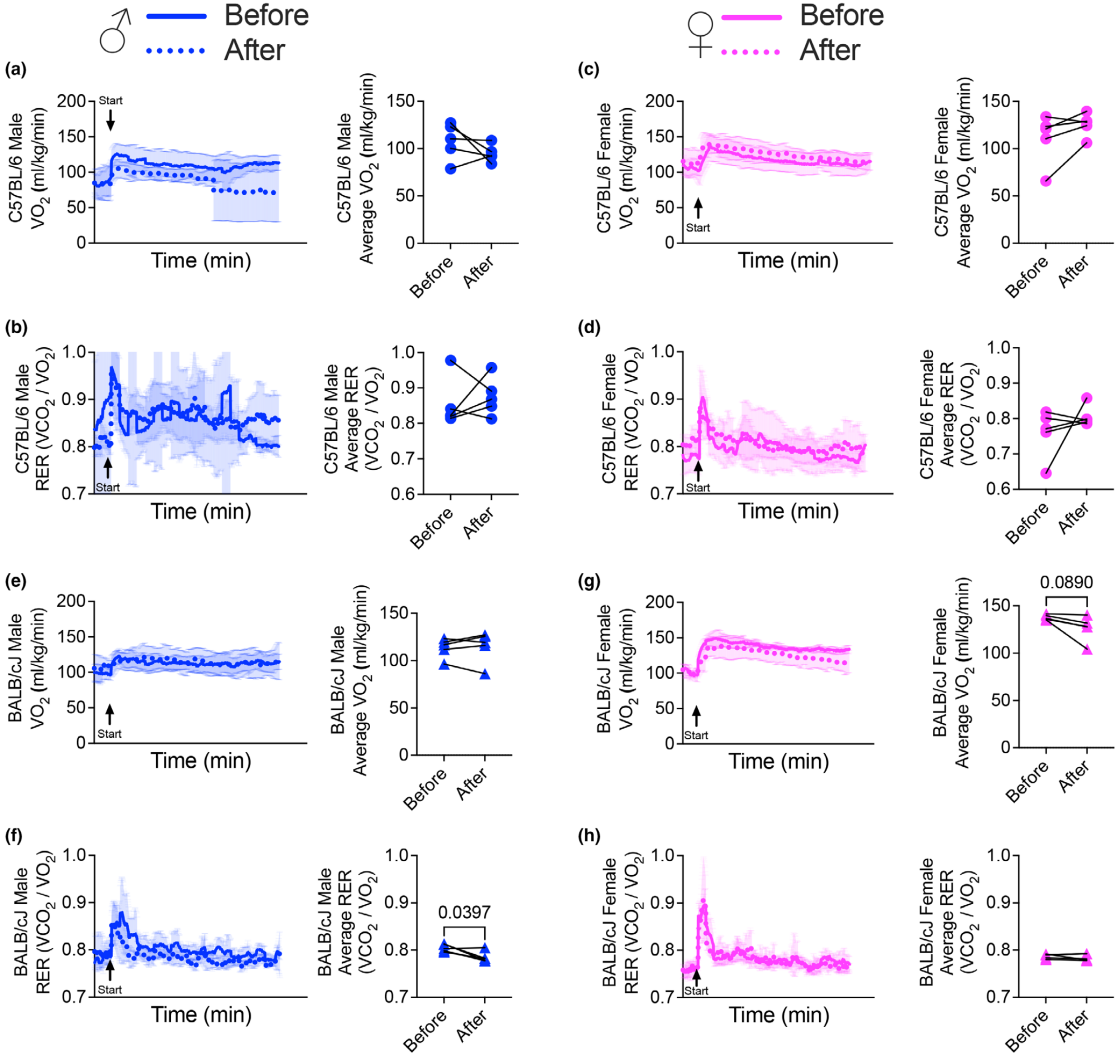
Effects of voluntary running on VO2 and RER during a progressive, sub‐maximal treadmill running test. Average VO2 (a, c) and respiratory exchange ratio (RER) (b, d) during a progressive, sub‐maximal treadmill running test in male and female C57BL/6 mice. Average VO2 (e, g) and respiratory exchange ratio (RER) (f, h) during a progressive, submaximal treadmill running test in male and female BALB/cJ mice. Values are presented as means ± SD.

Voluntary running for 4 weeks did not significantly alter average VO_2_, RER, or economy during a progressive, submaximal treadmill running test in either male or female C57BL/6 mice (Figure 4a–d; Figure S2A,B). In BALB/CJ mice, average VO_2_ and economy remained unchanged in males during the progressive submaximal test, while RER significantly decreased following training (Figure 4e–f; Figure S2D). Female BALB/CJ mice showed no significant differences in either VO_2_, RER, or economy after training (Figure 4g–h; Figure S2E). However, absolute submax VO_2_ did increase posttraining in female C57BL/6 and both sexes in BALBc/J mice (Figure S3A–D).

**FIGURE 5:**
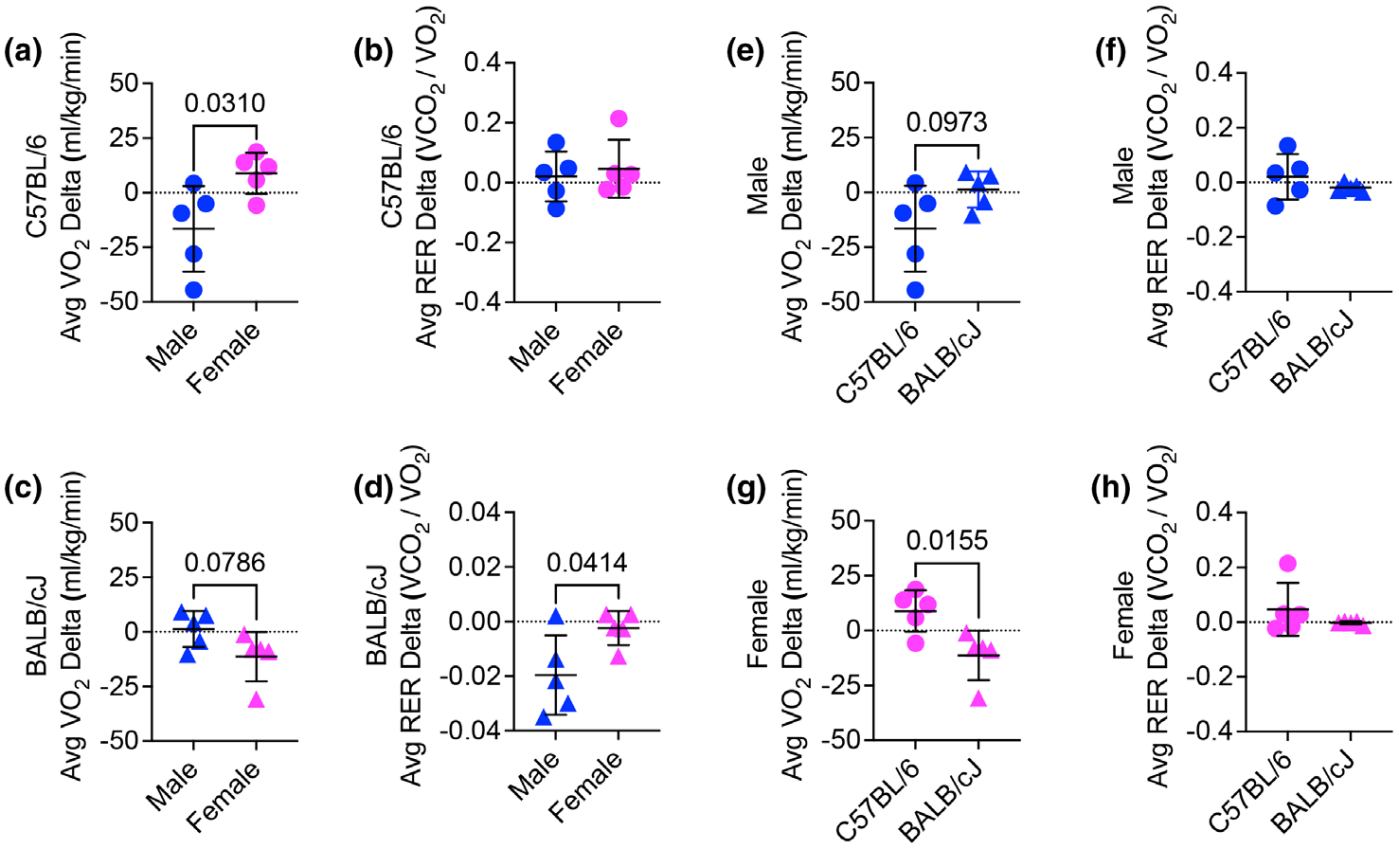
Differences in VO2 and RER during sub‐maximal treadmill running after the training period. (a) Comparison of VO2 delta between male and female C57BL/6 mice during sub‐maximal treadmill running after the training period (Student's *t*‐test). (b) RER delta during sub‐maximal treadmill running for male and female C57BL/6 mice (Student's *t*‐test). (c) Comparison of VO2 delta between male and female BALB/cJ mice during submaximal treadmill running after the training period (Student's *t*‐test). (d) RER delta during sub‐maximal treadmill running for male and female BALB/cJ mice (Student's *t*‐test). (e) VO2 delta comparison between male BALB/cJ and male C57BL/6 mice (two‐way repeated measures ANOVA). (f) RER delta between male BALB/cJ and male C57BL/6 mice (Student's *t*‐test). (g) VO2 delta comparison between female BALB/cJ and female C57BL/6 mice (Student's *t*‐test). (h) RER delta between female BALB/cJ and female C57BL/6 mice (Student's *t*‐test).

Compared to females, C57BL/6 male mice showed a significantly smaller VO_2_ delta in the progressive submaximal test (Figure 5a). However, there was no difference in RER delta between male and female C57BL/6 mice (Figure 5b). In BALB/cJ mice, males had a significantly lower VO_2_ delta compared to females (Figure 5c); however, running economy in females was improved posttraining (Figure S2F). No significant differences were observed between C57BL/6 and BALB/cJ males in either VO_2_ or RER delta during the submaximal test (Figure 5e,f), but there was a trend for the economy of C57BL/6 males to be improved compared to BALB/cJ males (Figure S2G). Despite large differences in running volume, BALB/cJ females had a significantly lower VO_2_ delta than C57BL/6 females (Figure 5g), in line with a significant improvement in running economy (Figure S2H). In females, no difference in RER delta was observed between the two strains (Figure 5h).

**FIGURE 6:**
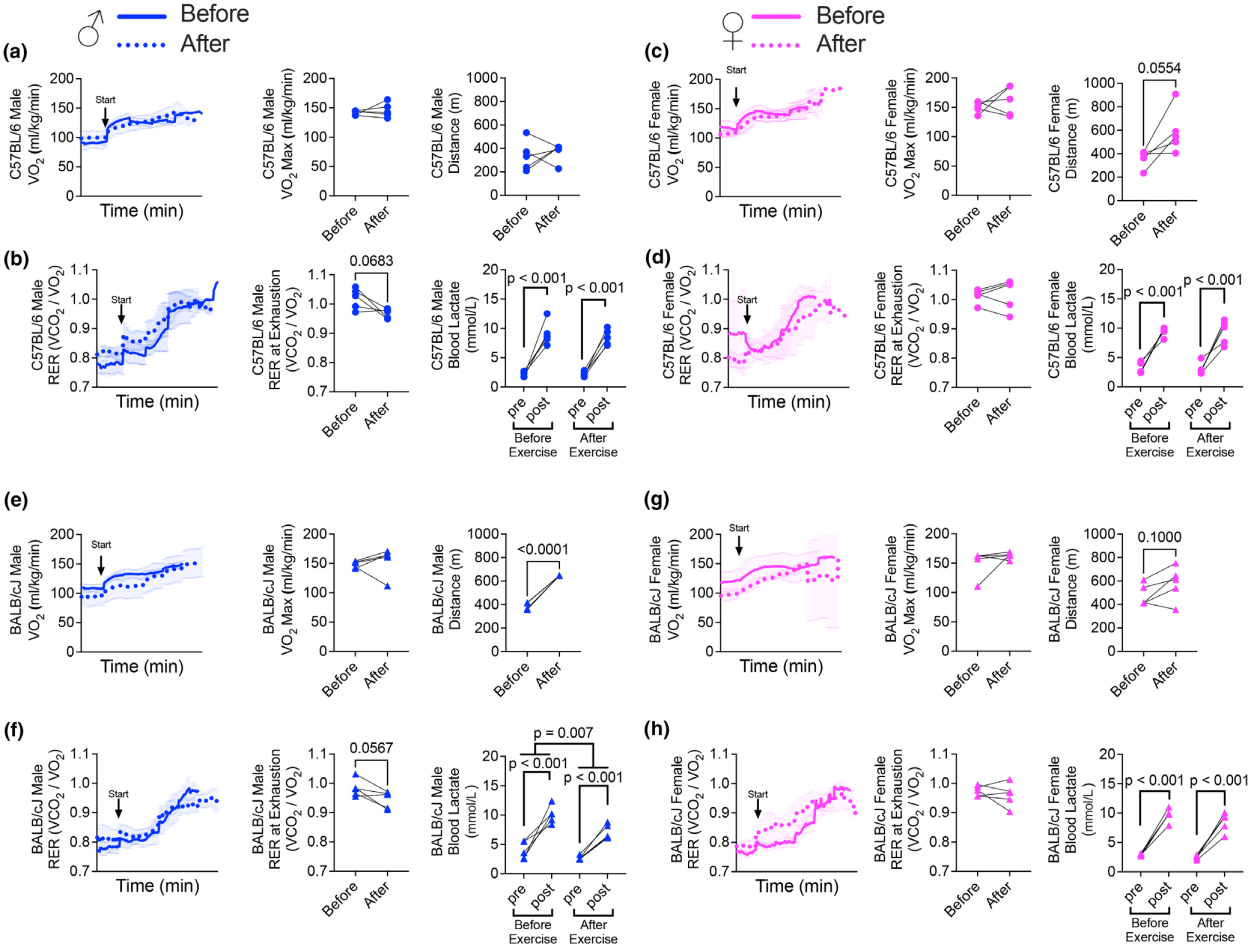
Effects of voluntary running on VO2 max, RER at exhaustion, endurance capacity, and blood lactate levels during a maximal treadmill running test. (a) VO_2_ max and distance in male C57BL/6 mice before and after the training period (Student's *t*‐test). (b) RER and lactate at exhaustion in male C57BL/6 mice before and after the training period (Student's *t*‐test). (c) VO_2_ max and distance in female C57BL/6 mice before and after the training period (Student's *t*‐test). (d) RER and lactate in female C57BL/6 mice before and after the training period (Student's *t*‐test). (e) VO_2_ max and distance in male BALB/cJ mice before and after the training period (Student's *t*‐test). (f) RER at exhaustion in male BALB/cJ mice before and after the training period (Student's *t*‐test). (g) VO_2_ max and distance in female BALB/cJ mice before and after the training period (Student's *t*‐test). (h) RER and lactate levels in female BALB/cJ mice before and after the training period (Student's *t*‐test).

Voluntary running did not significantly alter VO_2_ max or endurance capacity in male C57BL/6 mice, although RER at perceived exhaustion (confirmed via elevated blood lactate) displayed a trend to be lower after the training period (Figure 6a,b). Female C57BL/6 mice had no change in VO_2_ max or RER at exhaustion following the training period despite a trend (*p* = 0.0554) towards an increase in endurance capacity (Figure 6c,d). BALB/cJ male mice increased endurance capacity following the training period, which coincided with a trend (*p* = 0.0567) towards reduced RER and lower blood lactate at exhaustion, but independent of any change in VO_2_ max (Figure 6e,f). Female BALB/cJ mice showed no change in VO_2_ max, RER at exhaustion, or endurance capacity in response to a maximal treadmill running test after the training period. Absolute VO_**2**_max was increased after the training period in male Balb/c mice, and a trend was found in all others (Figure S3E–H).

**FIGURE 7:**
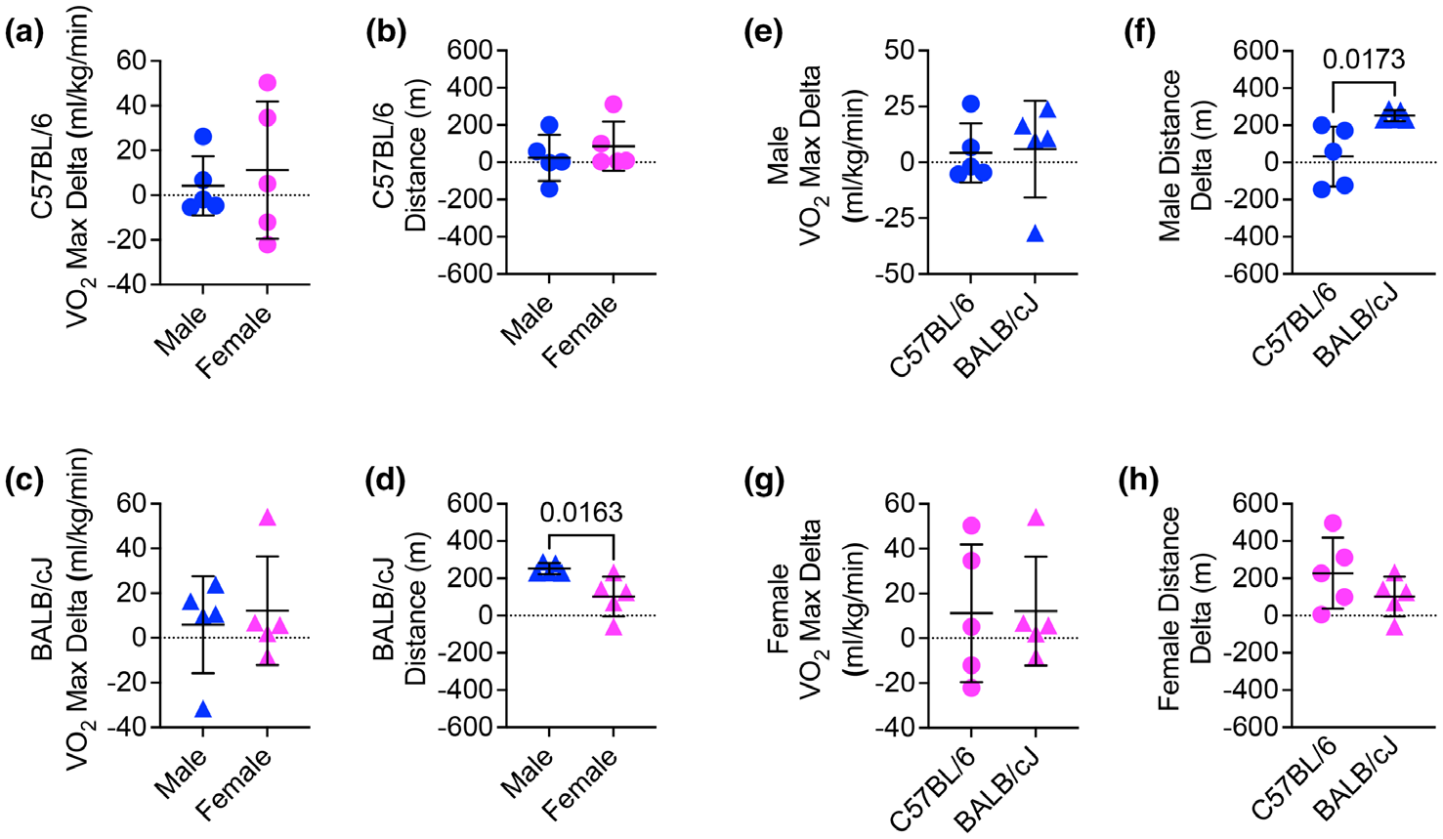
Delta changes in VO2 max and endurance capacity over the training period across strains and sexes. (a) VO_2_ max delta comparison between male and female C57BL/6 mice following the training period (Student's *t*‐test). (b) Endurance capacity delta comparison between male and female C57BL/6 mice (Student's *t*‐test). (c) VO_2_ max delta comparison between male and female BALB/cJ mice (Student's *t*‐test). (d) Endurance capacity delta comparison between male and female BALB/cJ mice (Student's *t*‐test). (e) VO_2_ max delta comparison between male BALB/cJ and male C57BL/6 mice (Student's *t*‐test). (f) Endurance capacity delta comparison between male BALB/cJ and male C57BL/6 mice (Student's *t*‐test). (g) VO_2_ max delta comparison between female BALB/cJ and female C57BL/6 mice (Student's *t*‐test). (h) Endurance capacity delta comparison between female BALB/cJ and female C57BL/6 mice (Student's *t*‐test).

Comparing delta change over the training period, we found no difference between male and female C57BL/6 mice in VO_2_max, absolute VO_2_max, or endurance capacity over the training period (Figure 7a,b, Figure S4). While there was no difference in VO_2_max delta between male and female BALB/cJ mice after the training period, male BALB/cJ mice had greater endurance capacity compared to females (Figure 7c,d). Male BALB/cJ endurance capacity delta was also greater than that of male C57BL/6 mice over the training period, though there was not a difference in VO_2_ max delta (Figure 7e,f). There was no difference in VO_2_ max or endurance capacity delta between females of the two strains (Figure 7g,h).

## 4 DISCUSSION

Both humans and rodents exhibit sex‐dependent differences in physical activity levels, body mass, body composition, and metabolism (Akerman et al., 2015; Lightfoot et al., 2004; Mauvais‐Jarvis, 2015). In the current study, we longitudinally investigated sex and strain‐specific relationships between voluntary running behavior and whole‐body adaptive responses. Within sex and strain, we found that differences in running behavior over 4 weeks did not uniformly predict pre/post changes to sub‐maximal or maximal VO_2_, despite changes in RER or maximal distance run that were consistent with anticipated adaptive responses to exercise training. In sum, our longitudinal results reinforce the notion that adaptations to exercise are sexually dimorphic and, in mice, VO2 measures may not be reflective of differences in voluntary running behavior across different strains.

Previous studies have shown voluntary running behavior varies between sex across different mouse strains (Lerman et al., 2002; Lightfoot et al., 2004). In studies comparing various strains, female C57BL/6 mice have been reported to voluntarily run between 5000 and 9000 m/day, whereas male C57BL/6 mice have been reported to run between 3000 and 7500 m/day (Konhilas et al., 2004; Lerman et al., 2002; Lightfoot et al., 2004). Increased voluntary running volume in female C57BL/6 mice has been shown to be maintained even as running volume declines with age (Konhilas et al., 2004). Our current findings in male C57BL/6 mice (~5000 m/day) are in line with the range noted above; however, we reported greater female running volume (~14,000 m/day), though the sexual dimorphic phenotype of female C57BL/6 mice running more than males was consistent. As the C57BL/6 strain has been shown to have a high propensity for voluntary running volume, we contrasted it with a strain with reportedly more moderate running volume behavior, BALB/cJ (Lerman et al., 2002; Lightfoot et al., 2004). Our current findings in male BALB/cJ mice (~9000 m/day) are higher than previously reported daily averages between 3500 and 6000 m/day (Lerman et al., 2002; Lightfoot et al., 2004), though both studies utilized a shorter voluntary running period of 2 or 3 weeks. In contrast to previous findings (Lerman et al., 2002), we found male BALB/cJ mice had elevated running volume compared to male C57BL/6 counterparts. Furthermore, we found an inverse running behavior between sexes in BALB/cJ mice compared to C57BL/6, with male BALB/cJ running significantly more than females, though female running volume was comparable to a previous study (Moore et al., 2023). Notably, female BALB/cJ running volume declined steadily over the 4‐week voluntary running period, which has been observed elsewhere (Turner et al., 2005). As the strain‐ and sex‐specific running volumes we observed were consistent with previous findings (Konhilas et al., 2004; Lerman et al., 2002; Lightfoot et al., 2004; Moore et al., 2023), we anticipated physiological assessments of adaptation to voluntary running (i.e. exercise testing) to be reflective of running volume.

Exercise training is associated with changes in body composition, such as reductions in fat mass and increases in lean mass (De Carvalho et al., 2016). Goh et al. showed 1 h of voluntarily running, 5 days a week, over a five‐month period in C57BL/6 mice was sufficient to lower fat mass and increase lean mass relative to sedentary counterparts (Goh & Ladiges, 2013). We did not observe any changes in lean mass or fat mass in male C57BL/6 or BALB/cJ mice, which may be due to the relatively short period (4 weeks) of voluntary running. Female C57BL/6 mice, however, increased lean mass and decreased fat mass, which may be due to the utilization of endogenous triglyceride stores in skeletal muscle. Holcomb et al. showed C57BL/6 sedentary females have higher skeletal muscle triglycerides, which decreased after endurance exercise, along with higher serum fatty acid concentrations (Holcomb et al., 2022). Thus, female C57BL/6 mice may have more accessible lipid sources and a greater capacity to mobilize them as a substrate to support exercise. Compared to male C57BL/6, female C57BL/6 mice had a trend for higher lean mass and significantly lower fat mass, which may be reflective of their increased running volume compared to males. However, there were no differences in body composition between female C57BL/6 and female BALB/cJ. Female BALB/cJ had an average running volume threefold lower than C57BL/6 females, suggesting that running volume is not related to strain‐dependent changes in body composition. However, males and females in both C57BL/6 and BALB/cJ strains gained body mass over the 4 weeks exposed to running wheels, suggesting follow‐up studies should prioritize weight stable animals.

During submaximal exercise in horses and recreationally active humans, VO_2_ increases over time (Thomas & Fregin, 1990; Westerlind et al., 1994). This phenomenon, known as VO_2_ drift, is generally associated with a decline in RER, reflecting a shift towards greater reliance on fat as an energetic substrate. In our study, although the submaximal protocol we used increased speed incrementally across the 90‐min test, we found that VO_2_ and RER stabilized after the initial 10‐min stage across all groups, regardless of sex, strain, or training status. Comparing delta changes between sexes within strain of average submaximal VO_2_ was generally not reflective of voluntary running behavior. Thus, VO_2_ and RER responses during submaximal running may not serve as sensitive metrics for evaluating voluntary run training‐induced adaptations in wild‐type C57BL/6 and BALB/cJ mice.

We calculated running economy before and after the training period from the 50 min section of the sub‐maximal test when treadmill running was constant at 19 m/min. While no pre/post differences in economy were noted, male C57BL/6 and female BALB/cJ had improved economy compared to the opposite sex within strain and to the same sex across strain. We have previously found that State 3 respiration in muscle from sedentary female B6SJL mice is similar to male B6SJL mice that increase State 3 respiration following 12 weeks of voluntary running (Brisendine et al., 2024). It is possible that mitochondria in sedentary female B6SJL mice are capable of respiring at their physiological maximum and, therefore, exercise does not result in further increases. By that same reasoning, our current findings may suggest that male C57BL/6 and female BALB/cJ mice possess a higher adaptive ceiling to 4 weeks of voluntary exercise training compared to their respective opposite sex.

VO_2_ max, the maximum rate of oxygen consumption during incremental exercise, is a common measure of aerobic capacity and endurance performance. Mice exhibit a linear relationship between running intensity and oxygen uptake, up to the point of exhaustion (Ayachi et al., 2016). We did not find an increase in VO_2_ max following 1 month of voluntary wheel running exercise in either strain or sex, which suggests no improvement in peak aerobic capacity. VO_2_max in untrained C57BL/6 mice between 6 and 12‐month‐old mice has been reported between approximately 130–180 mL/kg/min (Schaefer et al., 2022; Schefer & Talan, 1996). We have previously shown that mice on a C57BL6/J background had a VO_2_max of approximately 140 mL/kg/min following 8 weeks of voluntary wheel running, which was not different from sedentary counterparts (Guan et al., 2024). Therefore, our VO_2_max values of approximately 150 mL/kg/min for both C57BL/6 and BALB/cJ strains are reasonable compared to other studies and in response to exercise training. In mice, VO_2_max adaptations depend not just on the duration of the training period but also on intensity and cumulative work performed (Lerman et al., 2002). Kemi et al. utilized a forced treadmill protocol where mice ran for 2 h/day, 5 days/week at intervals set to approximately 85%–90% of baseline VO_2_max. This paradigm resulted in a 49% and 29% increase in C57BL/6 female and male VO_2_max, respectively (Kemi et al., 2002). However, male BALB/C and possibly female C57BL/6 mice showed increased distance run, which we and others have used as an indicator of increased exercise capacity following voluntary wheel or treadmill exercise training (Allen et al., 2025; Brisendine et al., 2024; Drake, Wilson, Cui, et al., 2021; Guan et al., 2024; Laker et al., 2017). Also, distance run during the VO_2_ max test was reflective of running behavior in male mice across strains and between males and females in BALB/C mice. In sum, voluntary running may not exert the progressive overload needed to increase VO2max compared to forced running protocols. Therefore, an increase in distance run may be a more appropriate performance metric following voluntary wheel running training paradigms.

Our study has several weaknesses that should be considered. Mice in our study gained body mass over the course of the study; therefore, it is possible our results are influenced by age‐related developmental changes. For example, excluding the change in body weight by calculating absolute VO_2_ (mL/h) during both submaximal and maximal treadmill protocols shows an increase following the training period in male BALB/cJ and female mice from both strains. Additionally, C57BL/6 mice ran an equivalent amount during both the light and dark cycles, suggesting a possible circadian disruption that could influence our results. However, this may not be that abnormal, as a recent study tracking daily activity in C57BL/6 mice for approximately 1.5 years noted a large variance in night activity, with night activity accounting for only 60% of activity on average (Pernold et al., 2021). Despite these noted weaknesses, our longitudinal design offers a distinct advantage by capturing dynamic changes within individual mice over time, rather than static intergroup comparisons. This design allowed us to capture within‐subject changes over time and better account for individual variability in training responsiveness. However, it may also explain why some of our findings, particularly related to VO_2_ max, RER, and distance to exhaustion—differed from those studies comparing to sedentary counterparts (König et al., 2020; Lerman et al., 2002).

In sum, our results suggest that voluntary running volume alone is not a definitive predictor of changes in aerobic adaptation or metabolic substrate utilization, as indicated by metabolic cage assessments during submaximal or maximal running. Finally, VO_2_ max may not be the best metric for the adaptive response to voluntary wheel running exercise paradigms, particularly if less than 4 weeks in nonweight stable mice.

## FUNDING INFORMATION

This work was not supported by extramural funds.

## ETHICS STATEMENT

All experiments and procedures were approved by the Institutional Animal Care and Use Committee (IACUC) at Virginia Tech.

## Supporting information


Data S1.
