# Supplementary material for: Sex differences in voluntary running behavior between C57BL/6 and BALB/cJ mouse strains do not correspond to changes in VO2 and RER
Source: Physiol Rep. 2025 Oct 10;13(19):e70604. doi: 10.14814/phy2.70604 (PMC12513855; doi:10.14814/phy2.70604)
Supplement: Supplementary file 1 — Data S1. [file PHY2-13-e70604-s001.docx]

**Supplementary Figures**

**Figure S1.** ***Day and night voluntary running distances in C57BL/6 and BALB/CJ mice during four weeks of wheel access****.* Average daily distance (meters) covered during the light (day) and dark (night) cycles was measured in male and female C57BL/6 (A, B) and BALB/cJ (C, D) mice over a 4-week voluntary wheel running period. Data analyzed via Student’s paired t-test.

**Figure S2.** ***Effect of running economy at 19 m/min in C57BL/6 and BALB/cJ mice.*** Average oxygen consumption (VO₂; mL/kg/m) was measured during the 50-minute steady-state phase at 19 m/min to assess running economy before and after one month of voluntary wheel running. (A)  C57BL/6 males, (B) C57BL/6 females. (C) Delta economy (post-training minus pre-training values) between sexes in C57BL/6 mice. (D)  BALB/cJ males, (E) BALB/cJ females. (C) Delta economy (post-training minus pre-training values) between sexes in BALB/CJ mice. **(**G) Delta economy between C57BL/6 and BALB/cJ males. **(**H) Delta economy between C57BL/6 and BALB/cJ females. Data analyzed via paired or unpaired t-tests as appropriate.

**Figure S3. Absolute VO₂ during submaximal and maximal treadmill exercise in C57BL/6 and BALB/cJ mice.** (A-D) Average absolute VO_2_ during submaximal exercise (paired Student’s t-test). (E-H) Absolute VO_2_max (paired Student’s t-test). Values are presented as mean ± SD.

***Figure S4 – Absolute VO2 delta during submax and max* treadmill exercise in C57BL/6 and BALB/cJ mice.** *(A) Average submax absolute VO₂ delta comparison between male and female C57BL/6 mice following the training period (Student’s t-test). (B) Average submax absolute VO₂ delta comparison between male and female BALB/cJ mice (Student’s t-test). (C) Average submax absolute VO₂ delta comparison between male C57BL/6 and BALB/cJ mice (Student’s t-test). (D) Average submax absolute VO₂ delta comparison between female C57BL/6 and BALB/cJ mice (Student’s t-test). (E) Absolute VO₂ max delta comparison between male and female C57BL/6 mice (Student’s t-test). (F) Absolute VO₂ max delta between male and female BALB/cJ mice (Student’s t-test). (G) VO₂ max delta comparison between male BALB/cJ and C57BL/6 mice (Student’s t-test). (H) VO₂ max delta comparison between female BALB/cJ and C57BL/6 mice (Student’s t-test).*
